# Use of Pristinamycin for Macrolide-Resistant *Mycoplasma genitalium* Infection

**DOI:** 10.3201/eid2402.170902

**Published:** 2018-02

**Authors:** Tim R.H. Read, Jørgen S. Jensen, Christopher K. Fairley, Mieken Grant, Jennifer A. Danielewski, Jenny Su, Gerald L. Murray, Eric P.F. Chow, Karen Worthington, Suzanne M. Garland, Sepehr N. Tabrizi, Catriona S. Bradshaw

**Affiliations:** Melbourne Sexual Health Centre, Alfred Health, Carlton, Victoria, Australia (T.R.H. Read, C.K. Fairley, M. Grant, E.P.F. Chow, K. Worthington, C.S. Bradshaw);; Monash University, Melbourne, Victoria, Australia (T.R.H. Read, C.K. Fairley, G.L. Murray, E.P.F. Chow, C.S. Bradshaw);; Statens Serum Institut, Copenhagen, Denmark (J.S. Jensen);; Murdoch Children’s Research Institute, Parkville, Victoria, Australia (J.A. Danielewski, J. Su, G.L. Murray, S.M. Garland, S.N. Tabrizi);; Royal Women’s Hospital, Parkville (J.A. Danielewski, J. Su, G.L. Murray, S.M. Garland, S.N. Tabrizi);; University of Melbourne, Parkville (S.M. Garland, S.N. Tabrizi, C.S. Bradshaw)

**Keywords:** pristinamycin, antimicrobal resistance, Mycoplasma genitalium, nongonococcal urethritis, pelvic inflammatory disease, bacteria, sexually transmitted infections, Australia

## Abstract

High levels of macrolide resistance and increasing fluoroquinolone resistance are found in *Mycoplasma genitalium* in many countries. We evaluated pristinamycin for macrolide-resistant *M. genitalium* in a sexual health center in Australia. Microbiologic cure was determined by *M. genitalium–*specific 16S PCR 14–90 days after treatment began. Of 114 persons treated with pristinamycin, infection was cured in 85 (75%). This percentage did not change when pristinamycin was given at daily doses of 2 g or 4 g or at 3 g combined with 200 mg doxycycline. In infections with higher pretreatment bacterial load, treatment was twice as likely to fail for each 1 log_10_ increase in bacterial load. Gastrointestinal side effects occurred in 7% of patients. Pristinamycin at maximum oral dose, or combined with doxycycline, cured 75% of macrolide-resistant *M. genitalium* infections. Pristinamycin is well-tolerated and remains an option where fluoroquinolones have failed or cannot be used.

*Mycoplasma genitalium* is a sexually transmitted bacterium and an established cause of urethritis, cervicitis, pelvic inflammatory disease, and obstetric complications (*1*,*2*). Azithromycin is frequently used alone or in combination with other antimicrobial drugs to treat these syndromes because of its activity against *Chlamydia trachomatis* and its long tissue half-life, enabling single-dose administration. European, US, and Australian treatment guidelines recommend azithromycin for treatment of *M. genitalium* infections (*3*–*6*). However a recent meta-analysis revealed the proportion of infections cured by azithromycin fell from 85% (95% CI 82%–88%) before 2009 to 67% (95% CI 57%–77%) during 2009–2013 (*7*). 

*M. genitalium* lacks a cell wall and is difficult to culture, hindering study of its antimicrobial susceptibilities and resistance mechanisms, but single-nucleotide substitutions in domain V of 23S rRNA do confer resistance to macrolides (*1*). In 2016, the prevalence of macrolide resistance mutations (MRM) in *M. genitalium* infections was 40%–60% in studies from Germany, Australia, Canada, and the United States (*8*–*11*). Recent work has demonstrated the selection of MRM during single-dose and multidose treatment with azithromycin (*11*–*13*).

Moxifloxacin is recommended for treating macrolide-resistant *M. genitalium* (*4*–*6*); however, fluoroquinolone resistance mutations associated with treatment failure recently have been reported in 15% of infected patients in Australia and 47% in Japan (*14*,*15*). Macrolide resistance exceeds 50% in *M. genitalium*–infected patients in Melbourne, and combined fluoroquinolone/macrolide resistance is found in 8.6%, rendering azithromycin and moxifloxacin ineffective in most of these cases (*11*,*16*). Moxifloxacin is also costly and not recommended during pregnancy and occasionally causes serious side effects (*17*).

Pristinamycin comprises 2 synergistic antimicrobial drugs: pristinamycin IA (a macrolide-like streptogramin B–type compound) and IIA (a streptogramin A–type compound) (*18*,*19*). Both bind to the 50S subunit of the bacterial ribosome causing bacteriostatic inhibition of protein synthesis, but the combination is bactericidal with a broad antibacterial spectrum that includes *Mycoplasma* spp. (*18*). Although mutations in *M. genitalium* have been associated with resistance to macrolides and fluoroquinolones, experience with pristinamycin for *M. genitalium* infections is limited, and the influence of mutations in the 23S and ribosomal genes on treatment efficacy are unknown. Mutations in the genes encoding ribosomal proteins L4 and L22 have been associated with in vitro resistance to pristinamycin and telithromycin, respectively, in *M. pneumoniae* (*20*) and have been described in cases of azithromycin treatment failure (*21*,*22*).

Based on favorable MICs (*23*) and early success in curing all 6 of 6 patients with dual macrolide and quinolone resistance (*12*), we evaluated pristinamycin during 2012–2016, initially in patients with *M. genitalium* infection that azithromycin and moxifloxacin failed to cure and then in patients in whom only azithromycin had failed, at Melbourne Sexual Health Centre (MSHC; Melbourne, VIC, Australia). We report the microbiological outcomes, and factors influencing these outcomes, for *M. genitalium* infections that were not cured by prior antimicrobial drug regimens and were treated with pristinamycin.

## Methods

Patients attending MSHC who have nongonococcal urethritis, pelvic inflammatory disease, cervicitis, or proctitis are routinely tested for *M. genitalium*, as are their sex partners. From August 2012 through November 2014, patients for whom azithromycin and moxifloxacin failed and for whom no other treatment options were available were treated with pristinamycin at a dose of 1 g 4 times/day for 10 days. Because of promising preliminary results and side effects data, in December 2014, pristinamycin became a second-line treatment after azithromycin failure. Two other pristinamycin regimens were evaluated during the study period: 1 g 2 times/day for 10 days and 1 g 3 times/day in combination with doxycycline. The pristinamycin/doxycycline combination was used based on evidence indicating this combination was effective in treating methicillin-resistant *Staphylococcus aureus* and theoretical and empirical evidence supporting antimicrobial drug combinations in several infections (*24*,*25*). 

Patients receiving pristinamycin from MSHC’s pharmacy were prospectively followed by a research nurse. The nurse extracted the following information and recorded it in a database: results of tests of cure, adverse effects, and posttreatment sex with an untreated or inadequately treated partner.

Patients were routinely advised to return for a test of cure 2–4 weeks after treatment. Microbiological cure was defined by a negative test for *M. genitalium* 14–90 days after start of treatment. Patients reporting posttreatment sex with an untreated partner (where the relationship preceded treatment) were excluded regardless of the result of their test of cure because of their high risk for reinfection. Patients who reported sex with treated partners or new partners were retained in the analysis and coded as being at risk for reinfection so that this information could be analyzed as a risk factor. The Alfred Hospital Ethics Committee approved the study (project 490/16).

### Laboratory Methods

We centrifuged urine (1 mL) at 10,000 × *g* for 10 min, discarded the supernatant, and resuspended the pellet in 0.2 mL of phosphate-buffered saline. Swabs were agitated in 0.4 mL phosphate-buffered saline to release cellular material. We extracted DNA from 0.2 mL of specimen on the MagNA Pure 96 Platform (Roche Applied Science, Mannheim, Germany) and stored it at −30°C. We used an *M. genitalium*–specific PCR targeting the 16S ribosomal RNA gene for detection and determined load by using a quantitative PCR targeting the MgPa gene (*12*,*26*). We performed partial sequencing of the 23S, L4, and L22 genes implicated in macrolide resistance (*21*) and ParC and GyrA genes indicated for fluoroquinolone resistance (*27*) on samples before and after treatment with pristinamycin. We sequenced PCR amplicons for each region in both directions by using Sanger sequencing (Australian Genome Research Facility, Melbourne, VIC, Australia) and performed sequence analysis by using the CLC Main Workbench version 7 (CLC Bio, Aarhus, Denmark). To identify mutations/sequence variations, we compared DNA sequences with the reference sequence *M. genitalium* G37 (GenBank accession no. NC_000908).

### Statistical Methods

We calculated proportions cured and 95% CIs using the binomial exact distribution and compared treatment subgroups by using Fisher exact test or the nonparametric trend test, where indicated. Bacterial load was log_10_ transformed, and the significance of comparisons was assessed by *t* test, paired when comparing before and after treatment samples from the same patient. We assessed the association between bacterial load and time since treatment by linear regression. We calculated odds ratios (ORs) and 95% CIs for predictors of treatment failure by logistic regression or exact logistic regression as appropriate, using Stata version 13 (StataCorp LLC, College Station, TX, USA).

## Results

**Figure 1:**
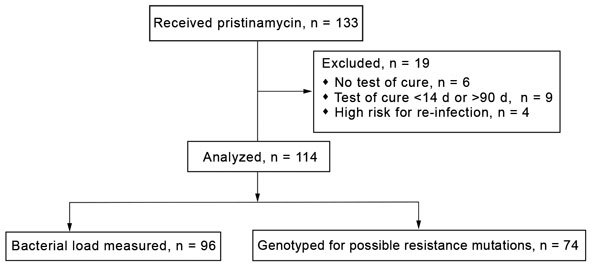
Selection of cases for analysis of microbiological cure of *Mycoplasma genitalium* infections with pristinamycin, Melbourne Sexual Health Centre, Melbourne, Victoria, Australia, 2012–2016.

During August 2, 2012–February 9, 2016, a total of 133 patients were treated with pristinamycin for *M. genitalium* infection. Patients were excluded from further study for 4 reasons: no test of cure (n = 6), test of cure <14 days from start of treatment (n = 4), test of cure >90 days from start of treatment (n = 5), and sex with untreated partners (n = 4) (Figure 1). The analysis comprised the remaining 114 patients. These patients had been unsuccessfully treated with >1 of the following antimicrobial drugs before pristinamycin: single-dose azithromycin (1 g; 34 [30%] patients); azithromycin 1.5 g (500 mg, then 250 mg/d for 4 d; 76 [67%]); moxifloxacin (400 mg/d for 10 d; 19 [17%]); or doxycycline (100 mg 2×/d for 7 d; 19 [17%]). Twenty-five (22%) patients had been treated with >2 courses of antimicrobial therapy. Four (4%) received pristinamycin as initial treatment because their partners had a resistant infection. 

Of the 114 patients in the analysis, data were available for 99 for analysis of bacterial load (before and after pristinamycin) and 74 for genetic sequencing of >1 region of interest. Median time to test of cure was 30 days (interquartile range 23–41).

### Characteristics of the Study Population

Of 114 patients treated with pristinamycin, 65 (57%) were men who have sex with men, 38 (33%) were heterosexual men, and 11 (10%) were women (Table 1). A first-pass urine sample was tested in 83 (73%) of the 114 patients; the remainder had rectal or cervical swab samples tested. The most common diagnosis was nongonococcal urethritis, present in 70 (61%) patients.

**Table 1:** Characteristics of patients with *Mycoplasma genitalium* infections treated with pristinamycin, Melbourne Sexual Health Centre, Melbourne, Victoria, Australia, 2012–2016

Characteristic	Men who have sex with men, n = 65	Heterosexual men, n = 38	Female, n = 11
Median age, y (interquartile range)	32.0 (27.4–37.3)	27.9 (24.6–34.6)	26.1 (22.6–28.2)
Sample, no.			
Urine*	39	38	6
Rectal swab	26	0	2
Cervical swab	Not applicable	Not applicable	3
Diagnosis, no.†			
Nongonococcal urethritis	37	33	
Proctitis	8	0	0
Pelvic inflammatory disease			2
Other‡	9	3	6
Asymptomatic, no.	11	2	3

*Includes 1 urethral swab sample. †Clinical diagnosis when first tested for *M. genitalium*. ‡Comprises urethral gonorrhea (n = 3), anal discharge (n = 3), other anal symptoms (n = 3), female dysuria (n = 2), vaginal discharge (n = 2), vaginal bleeding (n = 2), not recorded (n = 3).

### Microbiological Cure after Pristinamycin

Of 114 patients treated with any of the 3 pristinamycin regimens, infection was cured in 85 (75% [95% CI 66%–82%]). The proportion cured did not vary among the 3 regimens (p = 0.91) (Table 2). Proportions cured did not vary significantly by site of infection (urethral vs. anorectal), sex, or symptom status. Somewhat more asymptomatic infections were cured (94% [95% CI 70%–100%]) than symptomatic (71% [95% CI 61%–80%]; p = 0.07).

**Table 2:** *Mycoplasma genitalium* infections among 114 patients cured after 10 days of pristinamycin treatment, Melbourne Sexual Health Centre, Melbourne, Victoria, Australia, 2012–2016

Subgroup	Pristinamycin failure, no. (%)	Cured, no. (%, 95% CI)	p value*
Overall	29 (25)	85 (75, 66–82)	
Dosage regimen			
Pristinamycin 2 g/d	2 (22)	7 (78, 40–97)	0.91
Pristinamycin 3 g with doxycycline 200 mg/d	14 (26)	40 (74, 60–85)	
Pristinamycin 4 g/d	13 (25)	38 (75, 60–86)	
Site of infection			
Urethral infection, M	22 (29)	55 (71, 60–81)	0.20
Anorectal infection	4 (14)	24 (86, 67–96)	
Patient sex			
F	3 (27)	8 (73, 39–94)	1.0
M	26 (25)	77 (75, 65–83)	
Patient signs/symptoms			
Symptomatic	28 (29)	70 (71, 61–80)	0.07
Asymptomatic	1 (6)	15 (94, 70–100)	

*The 3 dosage regimens were compared by nonparametric test for trend. Fisher exact test used for other variables.

Sixty-eight (60%) patients had data recorded on medication adherence, and 7 (10%) had missed >1 dose. We found no difference in proportions cured between those who missed doses and those reporting 100% adherence (71% vs. 72%; p = 1.0). Data on reinfection risk were available for 92 (81%) patients; 5 (5%) were considered at risk for reinfection, but infections in all 5 were cured (p = 0.3).

### Effect of Bacterial Load on Microbiological Cure

**Figure 2:**
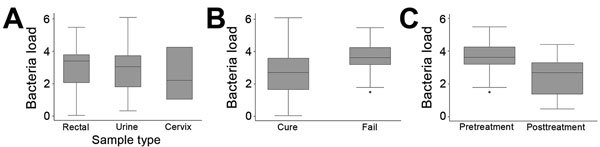
*Mycoplasma genitalium* bacterial loads (log_10_) and treatment outcomes, Melbourne Sexual Health Centre, Melbourne, Victoria, Australia, 2012–2016. A) *M. genitalium* load compared in urine (n = 67), rectal swab (n = 26), and cervical swab (n = 3) samples. For urine vs. rectal samples, p = 0.56; for urine vs. cervical samples, p = 0.70. B) Comparison of pretreatment *M. genitalium* loads in infections not cured (n = 26) and cured (n = 71) by pristinamycin. p<0.01. C) Comparison of *M. genitalium* loads in pretreatment and posttreatment samples from cases in which pristinamycin failed (n = 26). p<0.001..Box plots indicate 25th percentile (bottom of box), 75th percentile (top of box), median (horizontal line within box), and range (whiskers). Dots represent outlying individual observations.Dots under the error bars indicate individual outliers.

Mean pretreatment bacterial loads (log_10_) did not vary significantly among sample types for the 96 samples for which data were available: 2.9 log_10_ from 67 urine samples, 3.1 log_10_ from 26 rectal swab samples, and 2.5 log_10_ from 3 cervical swab samples (p = 0.56 for rectal vs. urine, p = 0.70 for cervical vs. urine) (Figure 2, panel A). We therefore analyzed the effect of bacterial load on treatment outcome in all samples. Mean *M. genitalium* load in pretreatment samples was 0.92 log_10_ higher in the 26 patients in whom pristinamycin failed (3.6 [95% CI 3.2–4.0] log_10_) than in the 71 in whom infection was cured (2.7 [95% CI 2.4–3.0 log_10_]; p<0.01) (Figure 2, panel B). For each log_10_ increase in bacterial load in pretreatment samples, the OR for treatment failure was 1.9 (95% CI 1.3–2.9;, p<0.01) (Table 3). In the 26 cases of pristinamycin failure for which paired samples were available, the mean bacterial load was significantly lower in posttreatment samples (2.3 [95% CI 1.8–2.7] log_10_) than in pretreatment samples (3.6 [95% CI 3.1–4.0] log_10_), a mean difference of 1.3 log_10_; p<0.001), indicating that unsuccessful treatment still reduced bacterial load (Figure 2, panel C). Posttreatment bacterial load did not vary with time to test of cure (p = 0.98). Restricting analyses to urine samples did not change any associations with bacterial load.

**Table 3:** Characteristics associated with pristinamycin failure in *Mycoplasma genitalium* infections, Melbourne Sexual Health Centre, Melbourne, Victoria, Australia, 2012–2016*

Characteristic	Cured, no. (%)	Failure, no. (%)	Unadjusted OR for failure (95% CI)	p value	Adjusted OR for failure (95% CI)	p value
Symptom						
Asymptomatic	15 (94)	1 (6)	Reference		Reference	
Symptomatic	70 (71)	28 (29)	6.0 (0.8–47.6)	0.09	4.1 (0.5–35.9)	0.20
Adherence to treatment						
Missed no doses	44 (72)	17 (28)	Reference			
Missed any doses	5 (71)	2 (29)	1.0 (0.2–5.9)	0.97		
No. antimicrobial drugs before pristinamycin						
0–1	62 (73)	23 (27)	Reference			
>2	20 (80)	5 (20)	0.7 (0.2–2.0)	0.48		
Male sexuality						
Men who have sex with men	52 (80)	13 (20)	Reference			
Heterosexual	25 (66)	13 (34)	2.1 (0.8–5.1)	0.11		
Bacterial load, all samples†	NA	NA	1.9 (1.3–2.9)	<0.01	1.9 (1.2–2.9)	<0.01
23S known macrolide resistance mutation‡						
Wild-type	6 (100)	0	Reference			
Mutation at 2058 or 2059	36 (67)	18 (33)	3.9 (0.52–∞)	0.17§		
Excluding wild-type cases						
Position 2059	22 (73)	8 (27)	Reference			
Position 2058	14 (58)	10 (42)	2.0 (0.6–6.2)	0.25¶		
23S G2162T‡						
Absent	21 (66)	11 (34)	Reference			
Present	5 (83)	1 (17)	0.38	0.41		
23S T2185G‡						
Absent	23 (74)	8 (26)				
Present	3 (43)	4 (57)	3.8 (0.70–21.0)	0.12		
23S additional G between positions						
2212 and 2213‡						
Absent	24 (67)	12 (33)				
Present	2 (100)	0	NA	0.32		
23S G2362A‡						
Absent	23 (72)	9 (28)	Reference			
Present	3 (50)	3 (50)	2.6 (0.40–15.1)	0.29		
L4# A515G N172S						
Absent	17 (68)	8 (32)	Reference			
Present	4 (50)	4 (50)	2.1 (0.4–10.7)	0.36		
L22# C430T Q144 stop codon						
Absent	23 (68)	11 (32)	Reference			
Present	2 (50)	2 (50)	2.1 (0.26–16.9)	0.49		

*The L4 and L22 mutations result in amino acid changes. Two ribosomal L4 gene mutations were not analyzed because only 1 case was identified for each mutation C94T (P32S) and G166C (E56Q). NA, not applicable; OR, odds ratio. †OR for each log_10_ increase in pretreatment bacterial load in 67 urine samples, 26 rectal swab samples, and 3 cervical swab samples. ‡*Escherichia coli* numbering. 23S mutations at 2058 and 2059 are established causes of macrolide resistance. §Exact logistic regression. ¶p value for comparison of proportions cured with mutations at 2058 vs 2059, after exclusion of wild-type cases. #*M. genitalium* numbering based on reference genome accession number L43967.2.

### Effect of Resistance Mutations on Microbiological Cure

Gene sequencing results were available from pretreatment samples of 74 patients. Sixty were successfully sequenced for mutations in the 23S rRNA gene, 38 for mutations in the L22 ribosomal gene, 33 for mutations in the L4 ribosomal gene, and 43 for fluoroquinolone resistance mutations.

#### 23S Macrolide Resistance Mutations

Of the 60 samples sequenced for 23S mutations, 6 (10%) had wild-type sequences, 24 (40%) had a known mutation at the 2058 position, and 30 (50%) had a known mutation at the 2059 position (*Escherichia coli* numbering). In all 6 patients without 23S mutations, pristinamycin cured infection, whereas it cured infection in only 36 (67% [95% CI 53%–79%]; p = 0.17) of the 54 patients with 23S mutations. We found no significant difference in proportions cured between those with 2058 and 2059 mutations (58% vs. 73%; p = 0.26) (Table 3). Other mutations were not associated with specific treatment outcomes (Table 4).

**Table 4:** Proportions of *Mycoplasma genitalium* infections cured with and without silent mutations (no resulting amino acid change) in the L4 and L22 genes, Sexual Health Centre, Melbourne, Victoria, Australia, 2012–2016*

Mutation	No. cured/no. treated (%)†	Unadjusted odds ratio for cure (95% CI)	p value
L4 T327C			
Absent	19/28 (68)		
Present	2/5 (40)	0.32 (0.23–3.39)	0.23
L4 G429A			
Absent	17/24 (71)		
Present	4/9 (44)	0.33 (0.05–2.12)	0.16
L4 C438T ‡			
Absent	18/25 (72)		
Present	3/8 (38)	0.23 (0.030–1.65)	0.08
L4 C468T			
Absent	17/27 (63)		
Present	4/6 (67)	1.18 (0.14–15.2)	0.86
L4 G507A‡			
Absent	18/25 (72)		
Present	3/8 (38)	0.23 (0.030–1.65)	0.08
L4 C516T‡			
Absent	18/25 (72)		
Present	3/8 (38)	0.23 (0.030–1.65)	0.08
L22 G81A K27			
Absent	18/26 (69)		
Present	7/12 (58)	0.62 (0.12–3.34)	0.51
L22 C231T N77			
Absent	17/24 (71)		
Present	8/14 (57)	0.55 (0.11–2.73)	0.39
L22 C351T L117			
Absent	23/34 (68)		
Present	2/4 (50)	0.48 (0.032–7.56)	0.48

*M. genitalium numbering based on reference genome GenBank accession number L43967.2 †The L22 gene was sequenced in 38 infections and the L4 gene was sequenced in 33 infections. ‡These 3 mutations always occurred together.

#### L22 Ribosomal Gene Mutations

We identified 3 mutations in the 38 infections where the L22 ribosomal gene could be sequenced, but only 1 led to an amino acid change, introducing a stop codon at position Q144 and shortening the protein by 1 amino acid, and none were significantly associated with treatment failure (Table 3). Of the 57 patients for whom sequences were available for L22 mutations and for 23S mutations, L22 mutations were more often co-detected in samples with the 2058 mutation (65%) than in samples with the 2059 mutation (24%) or in 23S wild-type samples (60%) (p<0.01).

#### L4 Ribosomal Gene Mutations

Of 33 isolates sequenced, 9 mutations were found in the L4 ribosomal protein gene (Table 4), but only 3 led to an amino acid change (P32S, E56Q, and N172S). We found no significant association between any individual or collective L4 ribosomal mutations and treatment outcomes.

We found mutations associated with fluoroquinolone resistance in 8 (19% [95% CI 8%–33%]) of 43 sequenced isolates. As expected, they were not associated with pristinamycin failure (p = 0.61).

### Predictors of Pristinamycin Failure

Bacterial load was the only significant factor associated with pristinamycin failure in univariate analysis; adherence, sexuality, or number of prior antimicrobial drugs were not associated with failure (Table 3). Prior prescription of moxifloxacin, doxycycline, or both did not affect outcome. The presence of symptoms was not associated with bacterial load (p = 0.67), and when both symptoms and bacterial load were included in a multivariate analysis, bacterial load was the only significant predictor of failure (adjusted OR 1.9 [95% CI 1.2–2.9]; p<0.01).

### Adverse Events

Among the 60 patients treated with pristinamycin without doxycycline, side effects were not common: 3 patients reported diarrhea, 2 reported nausea, and 1 reported headache. Of 54 patients treated with pristinamycin and doxycycline, side effects also were uncommon: 4 reported nausea, vomiting, or abdominal pain, and 2 had candidiasis. Overall, 8 (7% [95% CI 3%–13%]) patients treated with either regimen experienced gastrointestinal side effects.

## Discussion

Our investigation found that pristinamycin cured *M. genitalium* infections in 75% of patients who were unsuccessfully treated with azithromycin; in 17% of this population, moxifloxacin had failed to cure infection. The effectiveness of pristinamycin was the same when given at a dose of 1 g 4 times/day as when given as 1 g 3 times/day in combination with doxycycline at 100 mg 2 times/day. Higher pretreatment bacterial load was associated with treatment failure; the odds of failure increased almost 2-fold with each 1 log_10_ increase in load. Failed treatment reduced bacterial load by a mean 1.3 log_10_. Almost all of this population had macrolide-resistant infections, precluding assessment of the effect of any 23S ribosomal gene mutation on treatment response, but the site of MRM (2058 vs. 2059) and ribosomal protein gene mutations were not associated with treatment outcomes. 

Pristinamycin was well-tolerated with a low rate (7%) of reported side effects, but in this large case series, it did not perform better than moxifloxacin (89% in a recent meta-analysis) for macrolide-resistant *M. genitalium* infection (*28*). Given the lack of alternatives, pristinamycin remains an option during pregnancy and in other situations where fluoroquinolones have failed or are contraindicated.

Our study has some limitations. Because it was an evaluation in a clinical service and not a trial with controls, the documentation of reinfection risk and treatment adherence is not as complete. Nevertheless, because previous treatments had failed and pristinamycin is not approved to treat *M. genitalium* infections, sexual health physicians at MSHC were asked to be vigilant for these factors, and when documented (60%–80% of records), neither reinfection risk nor treatment adherence proved significant. Further limitations are that the combination of doxycycline with the 3 g/day dose and the few patients who received 2 g/day limit our power to compare the efficacies of different doses.

The only other report of the efficacy of pristinamycin in patients with *M. genitalium* infection was a series of 6 patients at MSHC in whom azithromycin and moxifloxacin treatment had failed (*12*). These patients were treated with pristinamycin at a dose of 1 g 4 times/day for 10 days at MSHC in 2013, and all 6 infections were cured (100% [97.5% 1-sided CI 54%–100%]). In contrast, infections were cured in 75% of the 51 patients in our study who received this regimen. The initial 6 cures are consistent with 25% therapy failure in our study when the CIs are considered. 

Pristinamycin is listed as a third choice in the 2016 European guideline on *M. genitalium*, and our findings do not encourage any stronger recommendation (*5*). No oral alternatives to pristinamycin are available, but 1 case of *M. genitalium* was cured by 1 week of daily injected spectinomycin, which, if available, is a less convenient option (*29*).

Given initial treatment successes with pristinamycin and promising in vitro data, we did not expect treatment to fail in 25% of macrolide-resistant *M. genitalium* infections. The reasons for this percentage of failures are not clear and did not appear related to adherence or reinfection, but higher bacterial load was strongly associated with pristinamycin failure. This finding has been observed in studies of the closely related macrolides, which show that higher load infections are more likely to fail treatment and lead to detection of resistance mutations (*11*,*30*). Higher load infections might be more likely to harbor heterotypic resistance within a subpopulation of organisms.

Antimicrobial susceptibility testing at the Statens Serum Institute (Copenhagen, Denmark; J.S. Jensen, unpub. data) indicated for both macrolide-resistant (n = 17) and susceptible (n = 23) *M. genitalium* strains, MICs were all susceptible (defined as <1 mg/L) (*31*). However, these MICs were close to the expected breakpoint, so we used the maximum dose of 1 g 4 times/day for 10 days. Macrolide-resistant strains had significantly higher MIC_90_ (0.50 mg/L) for pristinamycin than susceptible strains (MIC_90_ 0.125 mg/L; p = 0.003) but remained within the susceptible range (*32*). MRM are known to confer resistance to the streptogramin B component of pristinamycin (*18*), and mutations in position 2058 in *M. genitalium* have been associated with a higher MIC against macrolides, such as solithromycin (*33*), and in *M. pneumoniae* with erythromycin (*34*). Collectively, our data showing all 6 patients with no 23S MRM were cured, the MIC data, and the finding that infections were cured in 58% of patients with 2058 mutations, compared with 73% with 2059 mutations (p = 0.26), may indicate that 23S mutations influence the efficacy of pristinamycin and that there may be a differential effect of 2058 versus 2059 mutations in *M. genitalium*; however, this possibility requires further study.

Of the L4 and L22 ribosomal gene mutations present, only 1 of each resulted in an amino acid change. The L4 mutations detected were not close to the L4 loop near position 69 (*M. genitalium* numbering, position 66 *E. coli* numbering) known in other bacteria to cause macrolide resistance. By in vitro selection of resistance with subinhibitory concentrations of pristinamycin in the closely related *M. pneumoniae*, variable numbers of G insertions in position G60 (G59 in *M. genitalium*) was the only mechanism identified for pristinamycin resistance. These insertions occurred after a high number of passages suggesting a low potential for selection of resistance with pristinamycin (*20*). We identified no similar mutations in our dataset. The combination of doxycycline and pristinamycin was also no more effective than pristinamycin monotherapy, despite in vitro data suggesting a small additional effect of the combination, although not true synergy (J.S. Jensen, unpub. data).

We do not have an effective, safe, inexpensive oral drug for treating *M. genitalium* now that macrolide resistance is so prevalent. Fluoroquinolones are potentially toxic and vulnerable to increasing resistance. As our results indicate, pristinamycin cures 75% of macrolide-resistant infections with a course of up to 80 tablets; its price and availability vary around the world, but it may play a role where quinolones are contraindicated or ineffective. Two findings are consistent across several studies: 1) infections with lower bacterial loads are more likely to be cured (even in the presence of resistance) and 2) failed treatment with pristinamycin and azithromycin reduces bacterial load (*11*). These data highlight the urgent need for further work to determine the activity of new and existing antimicrobial drugs, and combinations of antimicrobial drugs, against this neglected infection.
